# Salidroside Ameliorates Radiation Damage by Reducing Mitochondrial Oxidative Stress in the Submandibular Gland

**DOI:** 10.3390/antiox11071414

**Published:** 2022-07-21

**Authors:** Yue-Mei Sun, Xin-Yue Wang, Xin-Ru Zhou, Chong Zhang, Ke-Jian Liu, Fu-Yin Zhang, Bin Xiang

**Affiliations:** 1 Laboratory of Oral and Maxillofacial Disease, Second Hospital of Dalian Medical University, Dalian 116023, China; ymsun163@163.com (Y.-M.S.); xywang@dmu.edu.cn (X.-Y.W.); zhouxr0210@163.com (X.-R.Z.); rush914@163.com (C.Z.); 2 Stony Brook Cancer Center, Renaissance School of Medicine, Stony Brook University, Stony Brook, NY 11794, USA; kliu@salud.unm.edu; 3 Department of Oral Surgery, Second Hospital of Dalian Medical University, Dalian 116023, China

**Keywords:** radiation damage, submandibular gland, oxidative stress, salidroside, mitochondrion

## Abstract

Radiotherapy for patients with head and neck cancer inevitably causes radiation damage to salivary glands (SGs). Overproduction of reactive oxygen species (ROS) leads to mitochondrial damage and is critical in the pathophysiology of SG radiation damage. However, mitochondrial-targeted treatment is unavailable. Herein, both in vitro and in vivo models of radiation-damaged rat submandibular glands (SMGs) were used to investigate the potential role of salidroside in protecting irradiated SGs. Cell morphology was observed with an inverted phase-contrast microscope. Malondialdehyde (MDA), superoxide dismutase (SOD), catalase (CAT), glutathione (GSH), mitochondrial ROS, mitochondrial membrane potential (MMP), and ATP were measured using relevant kits. The mitochondrial ultrastructure was observed under transmission electron microscopy. Cell apoptosis was determined by Western blot and TUNEL assays. Saliva was measured from Wharton’s duct. We found that salidroside protected SMG cells and tissues against radiation and improved the secretion function. Moreover, salidroside enhanced the antioxidant defense by decreasing MDA, increasing SOD, CAT, and GSH, and scavenging mitochondrial ROS. Furthermore, salidroside rescued the mitochondrial ultrastructure, preserved MMP and ATP, suppressed cytosolic cytochrome c and cleaved caspase 3 expression, and inhibited cell apoptosis. Together, these findings first identify salidroside as a mitochondrial-targeted antioxidant for preventing SG radiation damage.

## 1. Introduction

An estimated 830,000 new cases of head and neck cancer are reported worldwide each year [1]. Radiotherapy is an important treatment for 80% of patients with head and neck cancer [1]. Although significant progress has been made in radiotherapy modes, such as intensity-modulated radiotherapy, a considerable number of patients are still affected by the high rate of acute and late side effects. For example, radiation sialadenitis is a well-recognized severe complication [2]. Radiation sialadenitis mainly manifests as glandular swelling and pain, dry mouth, dysphagia, oral mucositis, and increased periodontal disease risk, all of which seriously impact the physical and mental health of patients [3]. Although amifostine has been identified as a radioprotector for salivary glands [4,5], it has strict indications and many side effects, such as nausea, hypotension, vomiting, allergic reaction, thrombocytopenia, and leucopenia; thus, its clinical application is not well tolerated by most patients [6]. In addition, pilocarpine, another radioprotector, can relieve xerostomia symptoms caused by radiotherapy [7], but it also causes some serious side effects, including excessive sweating, stomach upset, dizziness, frequent urination, worsening asthma, and fatigue [8]. Thus, it is essential to explore other safe and effective medications to prevent radiation damage in salivary glands for head and neck cancer patients receiving radiotherapy.

Ionizing radiation commonly leads to excessive reactive oxygen species (ROS) generation, which eventually contributes to the interruption of mitochondrial structure and function in many tissues and cells, such as skin, bone marrow mesenchymal stem cells, retinal precursor cells, and salivary gland cells [9,10,11,12]. Mitochondria are associated with numerous essential cellular functions, including ROS generation and scavenging, ATP production, intracellular Ca^2+^ signaling, and cell apoptosis [13]. However, few mitochondria-targeted treatments are available to protect irradiated salivary glands by reducing mitochondrial oxidative stress.

Salidroside (Sal) is the main component of *Rhodiola rosea* L., a Chinese herb that has been well documented for treating symptoms of stress, eliminating fatigue, improving physical activity, and alleviating high-altitude sickness [14]. Pharmacologically, salidroside exerts multiple activities, such as anti-inflammatory, antioxidation, and anti-apoptosis activity, in certain tissues and cells [15,16,17]. Importantly, salidroside has an antiradiation effect in human skin cells and umbilical vein endothelial cells [18,19]. Specifically, salidroside protects human keratinocyte cells (HaCaT) from ultraviolet B (UVB)-induced oxidative damage by upregulating Nrf2 translocation to the nucleus [18]; prevents UVB-induced premature senescence in human dermal fibroblasts via antioxidation and proinflammatory cytokine inhibition [20]; and protects human umbilical vein endothelial cells against γ-radiation by modulating antioxidase and ROS scavenging [19]. However, the effects of salidroside on irradiated salivary glands remain unknown.

In this study, we investigated the cytoprotective roles and the underlying mechanisms of salidroside on rat submandibular glands (SMGs) against X-ray ionizing radiation. Our findings demonstrated that salidroside protected SMGs from radiotoxicity at both the tissue and cell levels. Furthermore, the underlying mechanisms of salidroside antiradiation in SMGs were associated with its antioxidative and antiapoptotic roles along with mitochondrial ultrastructure and function maintenance, which led to the improved secretion function of SMGs. Because of the much reduced side effects and lack of toxicity of salidroside, as shown in various animal experiments as well as clinical trials [21], our findings suggest that salidroside could be used as a safe and effective mitochondrial-targeted radioprotectant to protect the salivary glands of head and neck cancer patients undergoing radiotherapy.

## 2. Materials and Methods

### 2.1. Cell Culture and Irradiation

The rat submandibular acinar cell line SMG-C6 was a gift from Dr. Cong (Peking University, Beijing, China). The cells were cultivated in Dulbecco’s modified Eagle’s medium (DMEM)/F12 (1:1 mixture) supplemented with 2.5% fetal bovine serum (FBS), 100 U/mL penicillin, 100 μg/mL streptomycin, 50 μg/mL gentamicin sulfate, 5 mM glutamine, 5 μg/mL insulin, 80 ng/mL epidermal growth factor, 1.1 μM hydrocortisone, 0.1 μM retinoic acid, 2 nM triiodothyronine, and 5 μg/mL transferrin in a humidified atmosphere with 5% CO_2_ at 37 °C. All reagents used for the cell cultivation experiments were obtained from Sigma-Aldrich (Sigma-Aldrich, St. Louis, MO, USA). SMG cells were irradiated with a single dose of X-ray radiation at 15 Gy as described previously [22], using an X-Rad 320 Irradiator (Precision X-ray, Inc., North Branford, CT, USA).

### 2.2. Cellular Morphologic Observation

Salidroside was purchased from Meilunbio (Dalian, China). SMG-C6 cells were pretreated with salidroside (5, 10, or 20 μM) for 24 h before irradiation and then observed with an inverted phase-contrast microscope (DMi1, Leica Microsystem, Germany) at 24, 48, and 72 h post-irradiation. The salidroside doses were selected as per a previous publication [18].

### 2.3. Cell Viability Assay

SMG-C6 cells were seeded in 96-well microplates at a concentration of 3 × 10^3^ cells/well and pretreated with salidroside (5, 10, or 20 μM) for 24 h before irradiation. Cell viability was assayed using a Cell Counting Kit-8 (CCK8, Dojindo, Kumamoto, Japan) in accordance with the manufacturer’s specifications. The absorbance was measured with a microplate reader (SpectraMax 190, Molecular Devices, Sunnyvale, CA, USA) at 450 nm at 24, 48, and 72 h after irradiation.

### 2.4. Examination of Antioxidant Parameters

SMG-C6 cells were pretreated with 10 μM salidroside for 24 h before irradiation. At 48 h post-irradiation, 1 × 10^6^ cells were incubated in lysis buffer for 10 min. After centrifugation for 10 min at 4 °C and 13,000× *g*, supernatants were collected. The malondialdehyde (MDA) levels were determined by the thiobarbituric acid reactive substances (TBARs) assay. Superoxide dismutase (SOD) activity was detected by the xanthine oxidase method. Catalase (CAT) activities and glutathione (GSH) levels were measured by colorimetric assay. All the above kits were purchased from KeyGEN Biotech (Nanjing, China).

### 2.5. Mitochondrial ROS Production Assay

SMG-C6 cells were treated as described above in Section 2.4 and mitochondrial ROS were detected with 5 μM MitoSOX™ red (Invitrogen, Carlsbad, CA, USA) in the dark at room temperature for 10 min, followed by washing three times in HBSS (with Ca^2+^ and Mg^2+^). Nuclei were stained for 5 min at room temperature with Hoechst (Thermo Fisher Scientific, Waltham, MA, USA). MitoTEMPO was used as a reference antioxidant. The images were observed under an inverted fluorescence microscope (Leica DMi3000 B, Mannheim, Germany) in 10 random microscopic fields at 400× magnification. The relative fluorescence intensity of MitoSOX™ red was analyzed by ImageJ software (Fiji, Version 1.0).

### 2.6. Transmission Electron Microscopy (TEM)

SMG-C6 cells were treated as described above in Section 2.4, fixed in 2.5% glutaraldehyde, and stained with 2% uranium acetate and 2.6% lead citrate. Then, sections of SMG-C6 cells were observed under TEM (JEM-2000EX) to assess the mitochondrial ultrastructure.

### 2.7. Mitochondrial Membrane Potential (MMP) Measurement

SMG-C6 cells were treated as described above in Section 2.4. The JC-10 assay (Sigma–Aldrich, St. Louis, MO, USA) was utilized at 48 h post-irradiation to detect the MMP of SMG-C6 cells. SMG-C6 cells were incubated with JC-10 dye loading solution in a 5% CO_2_ 37 °C incubator for 30 min. Carbonyl cyanide 4-(trifluoromethoxy) phenylhydrazone (FCCP; 10 μM) was treated for 20 min to serve as a positive control. The fluorescence was measured by a SpectraMax i3 Multi-Mode Microplate Reader (Molecular Devices, Sunnyvale, CA, USA) with an initial excitation and emission wavelength at 530/580 nm (red) and 485/530 nm (green), respectively. The red/green fluorescence intensity ratio indicates MMP level.

### 2.8. ATP Assay

SMG-C6 cells were treated as described above in Section 2.4 and analyzed at 48 h post-irradiation with an adenosine triphosphate (ATP) bioluminescence assay kit (Beyotime, Shanghai, China). Briefly, 2 × 10^5^ SMG-C6 cells were incubated on ice for 10 min in 200 μL ice-cold ATP detection lysate. The supernatant was collected after centrifugation at 12,000× *g* for 5 min at 4 °C. By using a standard curve, the luminescence was converted into ATP concentration with a SpectraMax i3 plate reader (Molecular Devices, Sunnyvale, CA, USA).

### 2.9. Isolation of Mitochondrial and Cytosolic Fraction

SMG-C6 cells were treated as described above in Section 2.4. Mitochondrial and cytosolic fractions of SMG-C6 cells were separated by differential centrifugation using a mitochondria isolation kit (Beyotime, Shanghai, China) in accordance with the manufacturer’s instructions. Cytosolic proteins were obtained for detecting the released cytochrome c.

### 2.10. Western Blot

SMG-C6 cells were treated as described above in Section 2.4. Cells were lysed, and total or cytosolic protein was extracted using the corresponding extraction buffer (Beyotime, Shanghai, China). Western blot analysis of cytosolic cytochrome c (host: rabbit; 1:2000 dilution, Proteintech, Wuhan, China), cleaved caspase 3 (host: rabbit; 1:1000 dilution, CST, MA, USA), and β-actin (host: mouse; 1:20,000 dilution, Proteintech, Wuhan, China) was performed following a standard procedure. Secondary antibodies were DyLight 680-labeled goat anti-rabbit and DyLight 800-labeled goat anti-mouse (Thermo Scientific, MA, USA). The bands were detected with an Odyssey system (LI-COR, Lincoln, NE, USA), and the image analysis was performed using ImageJ (Fiji, Version 1.0). β-actin was utilized as an internal standard.

### 2.11. Terminal Deoxynucleotidyl Transferase-Mediated dUTP Nick End Labeling (TUNEL) Staining

SMG-C6 cells were treated as described above in Section 2.4 and stained with the Cell Death Detection Kit (Beyotime, Shanghai, China) in accordance with the manufacturer’s specifications. Apoptotic cells were stained with green fluorescence in nuclei and examined in 10 random microscopic fields at 200× and 400× magnification under an inverted fluorescence microscope (Leica DMi3000 B, Mannheim, Germany).

The effect of salidroside on cell apoptosis in rat irradiated SMG tissues was further evaluated using TUNEL assay (Beyotime, Shanghai, China). Apoptotic cells were stained with brown nuclei. Ten different fields of each section were captured to count the number of apoptotic cells under light microscopy (Leica DM2000, Mannheim, Germany) at 400× magnification.

### 2.12. Animals and Irradiation

Wistar rats (male and female, weighing 180–230 g) were purchased from the animal center of Dalian Medical University and randomly separated into four groups (*n* = 3 for each sex in each group): control, salidroside alone (100 mg/kg, injected intraperitoneally with a previously reported dosage [17]), radiation alone (a single dose of 20 Gy X-ray at 2 Gy/min delivered by an X-RAD 320 Irradiator), and radiation with salidroside pretreatment (administered 1 h before radiation). SMG tissues were resected under 2.5% tribromoethanol anesthesia on post-irradiation Day 7. All the experimental procedures were approved by the ethical committee of Dalian Medical University (approval No. AEE21094) and followed the ARRIVE guidelines (https://arriveguidelines.org/arrive-guidelines accessed on 8 June 2021).

### 2.13. Hematoxylin-Eosin (HE) Staining

Rat SMG tissues were fixed in 10% neutral formalin and embedded in paraffin. The SMG sections were stained with hematoxylin and eosin (Solarbio, Beijing, China) and evaluated under light microscopy.

### 2.14. Measurement of Pilocarpine-Stimulated Saliva Flow

Pilocarpine-stimulated saliva secreted from rat SMGs was evaluated as previously reported [23]. In brief, saliva was collected for 10 min from surgically separated Wharton’s ducts from SMGs using a micropipette under 2.5% tribromoethanol anesthesia starting 5 min after pilocarpine stimulation.

### 2.15. Statistical Analysis

The data are presented as the mean ± SEM. One-way analysis of variance was performed to compare the differences between groups. The Bonferroni test was used for multiple comparisons. *p* < 0.05 was considered statistically significant. Graph analyses were performed in GraphPad PRISM 8.3.1 (GraphPad Software, San Diego, CA, USA).

## 3. Results

### 3.1. Salidroside Mitigated Radiation Damage in SMG Cells

**Figure 1:**
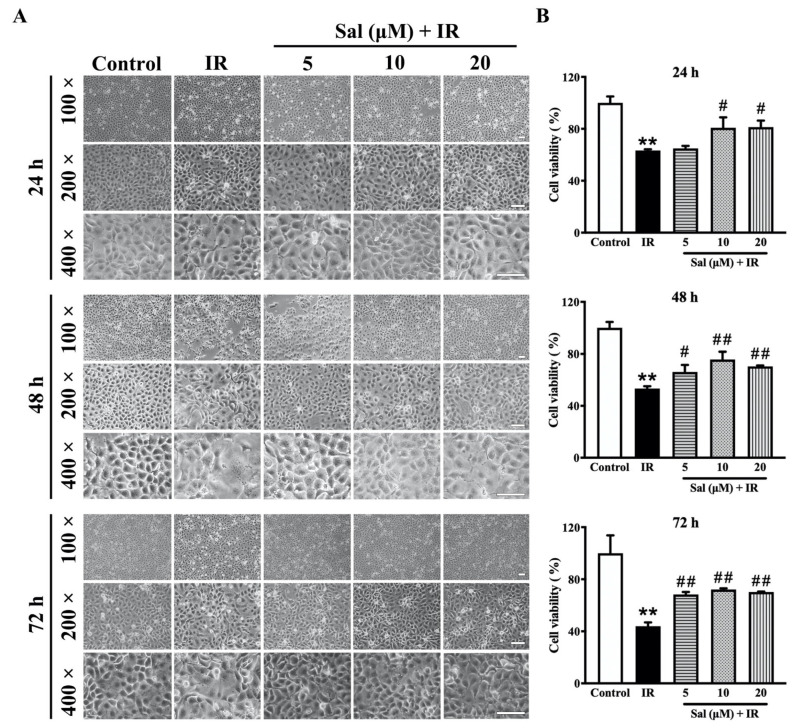
Salidroside mitigated radiation damage in SMG cells. SMG-C6 cells were used in (**A**) and (**B**). SMG-C6 cells were pretreated with salidroside (5, 10, or 20 μM) for 24 h before exposure to 15 Gy X-ray and tested at 24, 48, and 72 h after irradiation, respectively. (**A**) Representative images of SMG-C6 cells were obtained by an inverted microscope (bar = 100 μm). (**B**) Cell viability was detected with a CCK-8 assay. The data are presented as the mean ± SEM from 3 independent experiments, with each sample analyzed in triplicate. ** *p* < 0.01 vs. the control; # *p* < 0.05 and ## *p* < 0.01 vs. irradiation alone.

To investigate the cytoprotective potential of salidroside on irradiated salivary glands, SMG-C6 cells were observed under an inverted microscope. As shown in Figure 1A, normal SMG cells displayed a polygonal or oval shape and an orderly arrangement in the control group. After irradiation, the SMG cells became swollen, even with a dissolved cytomembrane, and presented a disordered arrangement at all time points. In contrast, when pretreated with salidroside, the cellular morphology and arrangement were similar to those of the control cells (Figure 1A), indicating that 10 μM salidroside exhibits the prominent antiradiation efficacy at 48 h post-irradiation.

Next, the viability of irradiated SMG-C6 cells with or without salidroside was examined with a CCK8 assay. After irradiation, the cell viability of the irradiated SMG-C6 cells was decreased by 35.7% (*p* < 0.01), 46.6% (*p* < 0.01), and 56.0% (*p* < 0.01) at 24, 48, and 72 h, respectively, in comparison with the controls (Figure 1B). However, treatment with 10 μM and 20 μM salidroside led to significant increases of 17.5% (*p* < 0.05) and 17.9% (*p* < 0.05) at 24 h, respectively; treatment with 5 μM, 10 μM, and 20 μM salidroside led to significant increases of 12.7% (*p* < 0.05), 22.3% (*p* < 0.01), and 17.1% (*p* < 0.01) at 48 h, respectively; and treatment with 5 μM, 10 μM, and 20 μM salidroside led to significant increases of 24.3% (*p* < 0.01), 28.1% (*p* < 0.01), and 26.3% (*p* < 0.01) at 72 h, respectively, compared with the irradiated alone group (Figure 1B). Taken together, these data demonstrate that salidroside protects SMG cells from radiation damage.

### 3.2. Salidroside Enhanced Antioxidant Defense and Reduced Mitochondrial ROS Generation in Irradiated SMG Cells

**Figure 2:**
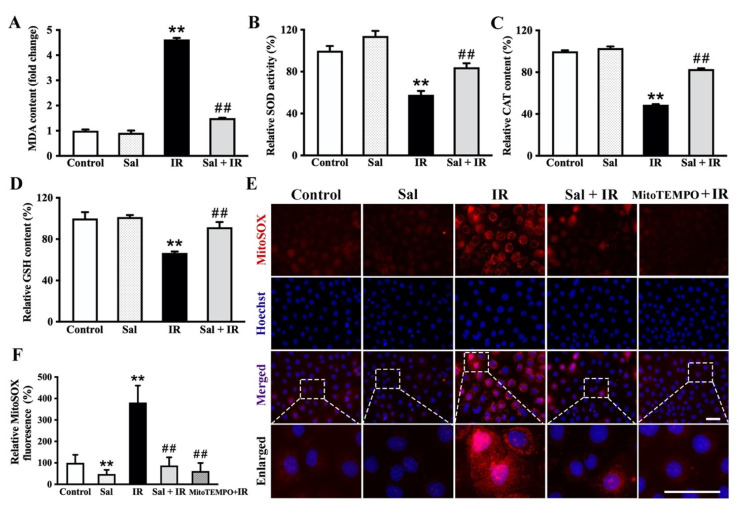
Salidroside enhanced antioxidant defense and reduced mitochondrial ROS in irradiated SMG cells. SMG-C6 cells were pretreated with 10 μM salidroside for 24 h before exposure to 15 Gy X-ray and detected after irradiation for 48 h. (**A**) MDA level, (**B**) relative SOD activity, (**C**) relative CAT content, and (**D**) relative GSH content were determined using the relevant kits. (**E**) Levels of mitochondrial ROS were detected by MitoSOX™ red (bar = 50 μm). Red fluorescence indicates positive staining. (**F**) Quantitative analysis of the mitochondrial ROS in (**E**). The data are presented as the mean ± SEM from 3 independent experiments, with each sample analyzed in triplicate. ** *p* < 0.01 vs. the controls; ## *p* < 0.01 vs. the irradiation alone.

Ionizing radiation commonly results in oxidative stress injury in salivary glands [24]. To evaluate the effects of salidroside on antioxidant defense in irradiated SMG cells, we measured the levels of MDA, CAT, and GSH as well as the activity of SOD using relevant kits. The MDA level was sharply increased by 3.6-fold (*p* < 0.01) in the irradiated alone group compared with the control group, but salidroside pretreatment mostly restored its value to the control level (Figure 2A). Conversely, radiation significantly reduced the levels of SOD, CAT, and GSH, which were decreased by 42.1% (*p* < 0.01), 51.1% (*p* < 0.01), and 33.3% (*p* < 0.01), respectively, compared with the controls (Figure 2B–D), while the salidroside pretreatment significantly increased the antioxidant levels in irradiated SMG cells, with SOD increased by 29.0% (*p* < 0.01), CAT by 34.0% (*p* < 0.01), and GSH by 24.9% (*p* < 0.01) compared with the irradiated alone group (Figure 2B–D). The MDA, SOD, CAT, and GSH levels in the salidroside alone group did not significantly differ in comparison with the controls (Figure 2A–D). These results demonstrate that radiation induces a strong oxidative stress response in SMG cells while salidroside protects SMG cells against oxidative damage.

Mitochondria have been widely reported to perform essential roles in regulating redox-dependent cellular processes through mitochondrial ROS production. To clarify the impact of salidroside on radiation-induced mitochondrial oxidative stress in the salivary gland, mitochondrial ROS levels in irradiated SMG-C6 cells were detected with MitoSOX™ red. Our results showed that radiation induced a marked increase in the production of mitochondrial ROS in SMG cells while salidroside significantly decreased the mitochondrial ROS to levels similar to the control level (Figure 2E,F). Collectively, these findings reveal that salidroside exerts antioxidant activities to ameliorate radiation damage by scavenging mitochondrial ROS in SMG cells.

### 3.3. Salidroside Alleviated Mitochondrial Ultrastructural Damage in Irradiated SMG Cells

**Figure 3:**
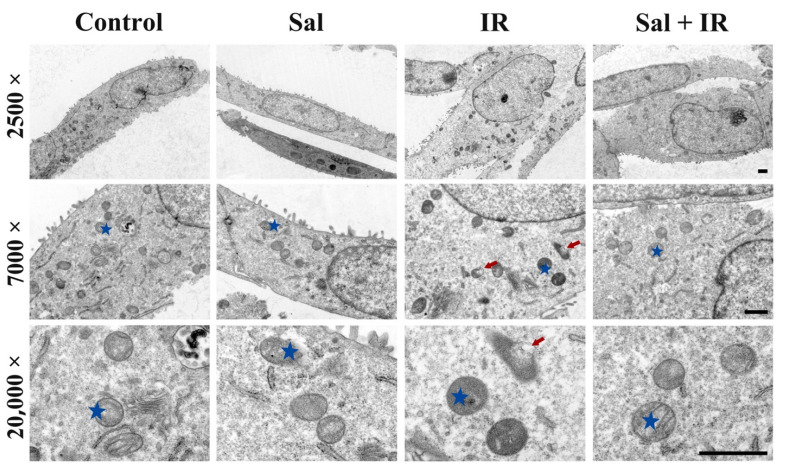
Salidroside rescued mitochondrial damage in irradiated SMG cells. SMG-C6 cells were pretreated with 10 μM salidroside for 24 h before irradiation and then evaluated with TEM 48 h post-irradiation. Representative images of SMG-C6 cells are shown (*n* = 3, for each group; bar = 1 μm). The asterisk indicates a healthy mitochondrion, and the arrow indicates a damaged mitochondrion.

To investigate the effect of salidroside on mitochondria in irradiated salivary glands, the mitochondrial ultrastructure in irradiated SMG cells was observed under TEM. The intact mitochondria in SMG cells were damaged by radiation, indicating that the mitochondrial inner and outer membranes turned indistinct and that the mitochondrial cristae were destroyed, as indicated by the red arrows in Figure 3-IR (7000× and 20,000× magnification). In contrast, the mitochondrial ultrastructure was rescued with salidroside pretreatment, which was similar to the controls (Figure 3-Sal + IR, 7000× and 20,000× magnification). Meanwhile, the mitochondrial ultrastructure was well maintained in the salidroside alone group and showed intact inner and outer membranes and distinct cristae (Figure 3-Sal). These findings provide first-hand proof that salidroside attenuates the radiation-induced mitochondrial damage in SMG cells.

### 3.4. Salidroside Maintained MMP and Enhanced ATP Production in Irradiated SMG Cells

**Figure 4:**
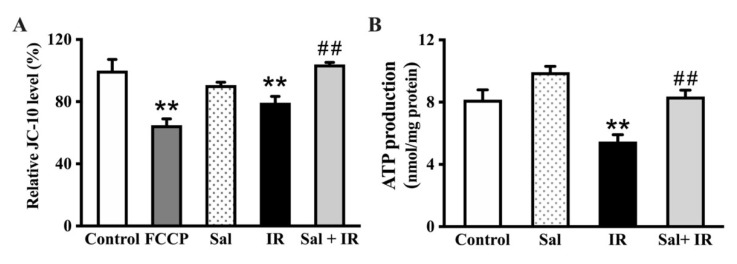
Salidroside maintained MMP and enhanced ATP in irradiated SMG cells. SMG-C6 cells were treated as described above in Figure 3. (**A**) MMP was detected using a JC-10 assay. FCCP was utilized as a positive control. (**B**) ATP levels were detected with an ATP assay kit. The data are displayed as the mean ± SEM from 3 independent experiments, with each sample analyzed in triplicate. ** *p* < 0.01 vs. the controls; ## *p* < 0.01 vs. the irradiation alone.

Next, we assessed the effect of salidroside on MMP in irradiated SMG-C6 cells utilizing a JC-10 assay. The ratio of JC-10 was markedly decreased by 21.7% (*p* < 0.01) in irradiated cells compared with the control group (Figure 4A), indicating the disruption of the MMP induced by radiation. However, salidroside pretreatment essentially restored MMP to the control level (Figure 4A), thus demonstrating that salidroside protects MMP from radiation damage in SMG cells.

Moreover, we examined the role of salidroside on ATP production in irradiated SMG-C6 cells using the ATP assay. X-ray radiation induced a significant reduction in ATP (approximately 2.7 nmol/mg protein (*p* < 0.01)) compared with the controls, whereas the intracellular ATP level was significantly increased by 2.9 nmol/mg protein (*p* < 0.01) in irradiated cells pretreated with salidroside (Figure 4B). Taken together, these findings further demonstrate that salidroside restores MMP and the energy metabolism of mitochondria in SMG cells after exposure to radiation.

### 3.5. Salidroside Inhibited Cellular Apoptosis by Suppressing Cytochrome c and Cleaved-Caspase 3 in Irradiated SMG Cells

**Figure 5:**
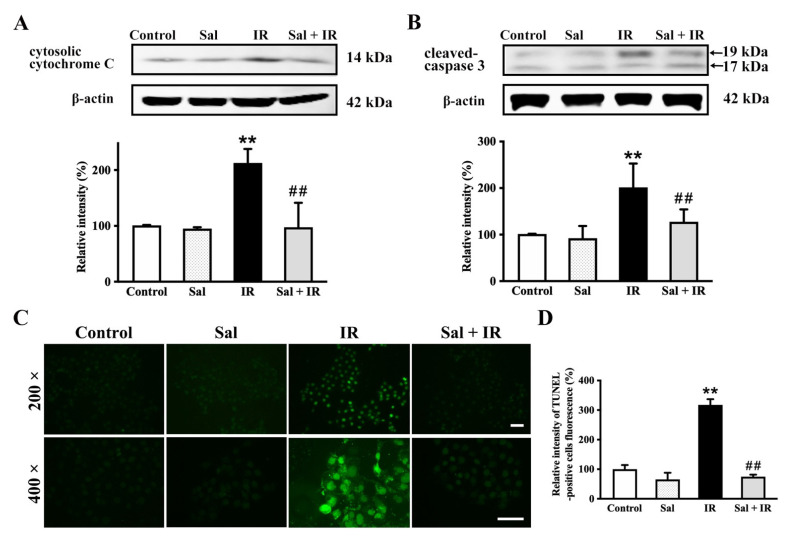
Salidroside inhibited cellular apoptosis by suppressing cytochrome c and cleaved-caspase 3 in irradiated SMG cells. SMG-C6 cells were treated as described above in Figure 3. (**A**) Release of cytochrome c and (**B**) cleaved caspase 3 were measured by Western blot. (**C**) Apoptosis was examined with a TUNEL assay. Nuclei of apoptotic cells were dyed with green fluorescence (bar = 100 μm). (**D**) Relative intensity of TUNEL-positive cell fluorescence. Data in (**A**,**B**,**D**) are the mean ± SEM (*n* = 3), with every data point assessed in triplicate. ** *p* < 0.01 vs. the controls. ## *p* < 0.01 vs. irradiation alone.

Decreased MMP commonly results in the opening of mitochondrial permeability transition pores, which leads to the release of cytochrome c from the mitochondria to the cytosol and the subsequent activation of proapoptotic caspases to induce cell apoptosis [25]. In this study, the expression levels of cytosolic cytochrome c and cleaved-caspase 3 were determined by Western blot. The release of cytosolic cytochrome c and the expression of cleaved-caspase 3 were significantly increased by 112.9% (*p* < 0.01) and 101.5% (*p* < 0.01) in irradiated cells, respectively, compared with the controls (Figure 5A,B). In contrast, salidroside significantly reduced both cytosolic cytochrome c release and cleaved caspase 3 expression by 115.3% (*p* < 0.01) and 73.9% (*p* < 0.01), respectively, compared with irradiation alone (Figure 5A,B).

Furthermore, the efficacy of salidroside on cell apoptosis in irradiated SMG cells was evaluated with a TUNEL assay. The nuclei in TUNEL-positive cells presented green fluorescence (Figure 5C). The relative fluorescence intensity of TUNEL-positive cells in the irradiated alone group was significantly increased by 217.5% (*p* < 0.01) compared with the controls, while that in the irradiated cells pretreated with salidroside was returned to the control value (Figure 5C,D). Taken together, these findings provide further evidence that salidroside inhibits apoptosis induced by radiation in SMG cells through the mitochondrial-related apoptotic signaling pathway.

### 3.6. Salidroside Alleviated Glandular Atrophy and Promoted Secretion Function in Irradiated SMG Tissues

**Figure 6:**
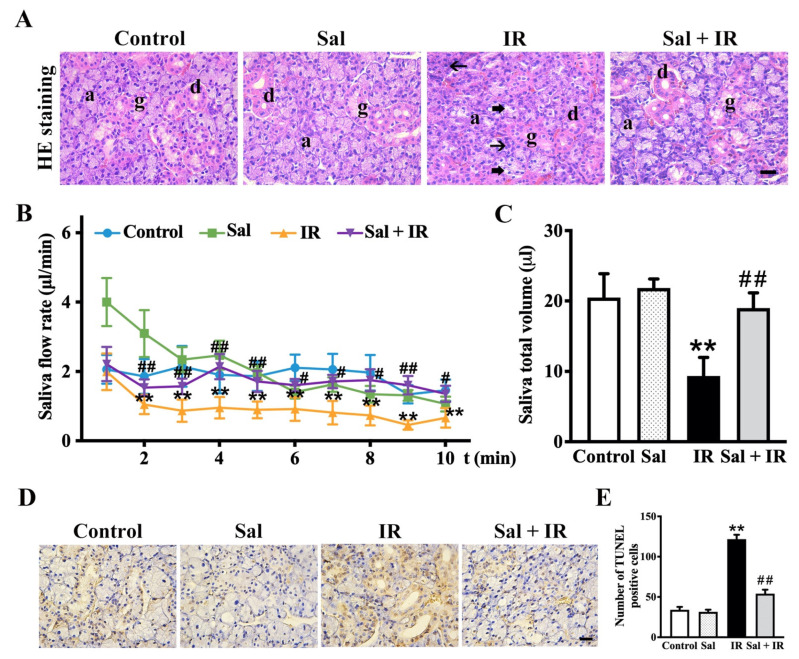
Salidroside alleviated radiation damage and promoted saliva secretion in rat SMG tissues. (**A**) Representative images of rat SMG tissues with HE staining (bar = 25 µm, *n* = 6 for each group). Thin arrows represent atrophy in acinar cells, and thick arrows represent vacuolar degeneration in GCT cells and acinar cells. Abbreviations: a, acinar cell; d, ductal cell; g, granular convoluted tubule cell. (**B**) Analysis of the saliva flow rate and (**C**) saliva bulk volume in pilocarpine-stimulated rat SMGs. The data are shown as the mean ± SEM (*n* = 6 for each group). (**D**) Representative images of apoptotic cells in irradiated SMGs (*n* = 6 for each group). Brown nuclei indicate positive staining (bar = 25 µm). (**E**) Analysis of the number of TUNEL-positive cells in (**D**). ** *p* < 0.01 vs. the controls. # *p* < 0.05 and ## *p* < 0.01 vs. irradiation alone.

To confirm the cytoprotective effect of salidroside on irradiated salivary glands using an in vivo model, rat SMG tissues were observed with HE staining. Histologically, as shown in Figure 6A, the acinar and granular convoluted tubule (GCT) cells displayed dramatic atrophy (thin arrows) and vacuolar degeneration (thick arrows) after irradiation. In contrast, the histomorphological structure of the irradiated SMGs was well maintained by salidroside (Figure 6A).

After pilocarpine stimulation, as shown in Figure 6B,C, the saliva flow rate and bulk volume were markedly decreased by radiation in SMG tissues while both were significantly increased by salidroside. Collectively, these data demonstrate that salidroside not only protects the histostructure of the irradiated SMGs but also restores the secretion function after irradiation.

To further elucidate the effect of salidroside on anti-apoptosis in rat irradiated SMG tissues, TUNEL assay was conducted. TUNEL-positive cells were dramatically increased after irradiation, whereas salidroside significantly reduced the number of apoptotic cells (Figure 6D,E), providing direct evidence that salidroside protects SMG from apoptosis after irradiation.

## 4. Discussion

Although studies have reported for several decades that the ultrastructure and functions of mitochondria in salivary glands are destroyed by ionizing radiation [12,26,27,28], mitochondria-targeted treatment options remain unavailable. The results of this study showed that salidroside protected the histostructure and secretion function of SMGs from radiation damage both in vivo and in vitro. Moreover, salidroside displayed strong antioxidant properties in irradiated SMGs by suppressing MDA and elevating SOD, CAT, and GSH. Importantly, salidroside improved the recovery of the mitochondrial ultrastructure and energy metabolism by scavenging mitochondrial ROS, which eventually led to the inhibition of cellular apoptosis in irradiated SMGs.

Cellular ROS are considered pivotal factors in radiation-induced damage in salivary glands. Our study and previous work have reported that cellular ROS levels markedly increase in salivary glands after irradiation both in vivo and in vitro [12,29,30]. Excessive production of ROS induces microvascular dysfunction, causes DNA damage, suppresses cell proliferation, injures stem/progenitor cells, destroys mitochondrial homeostasis, and triggers cell apoptosis in the salivary gland [12,29,31,32]. Recent studies have shown that Tempol (a membrane-permeable radical scavenger) and MitoTEMPO (a mitochondrial ROS scavenger) may serve as potential radioprotectors of salivary glands by diminishing cellular and mitochondrial ROS [31,33]. However, both Tempol and MitoTEMPO are chemical rather than medicinal. Notably, salidroside has drawn much attention as an attractive bioagent because of its significant antioxidant and antiradiation effects [19]. Salidroside scavenges ROS and alleviates DNA injury in human umbilical vein endothelial cells after irradiation [19]. Our results initially revealed that salidroside decreased MDA content and increased SOD activity and CAT and GSH contents in irradiated SMG cells. It is well known that MDA is the most frequently measured biomarker of oxidative stress [34], and SOD is an oxidoreductase enzyme that transforms superoxide radical (O_2_^•^^−^) to hydrogen peroxide (H_2_O_2_), which is then detoxified to H_2_O by CAT or GSH [35]. Levels of SOD, CAT, and GSH represent the antioxidant defense ability in mammalian cells. More importantly, salidroside significantly reduced mitochondrial ROS in SMG cells after irradiation, and the degree of the decrease was similar to that in the MitoTEMPO treatment group, indicating that salidroside exerts a mitochondrial-targeted antioxidant role in irradiated salivary glands.

As one of the main intracellular ROS sources, mitochondria are more vulnerable when exposed to radiation [36]. Excessive ROS damage mitochondria both in structure and function [12]. Consistent with our findings and the results of previous studies [12,26,27,28], the mitochondrial ultrastructure of SMG cells was destroyed by radiation. In contrast, the mitochondrial ultrastructure was well preserved by salidroside in irradiated SMG cells. Normally, solutes such as hydrogen ions cannot freely pass through the inner mitochondrial membrane (IMM) but are strictly governed by proton pumps (complexes I, III, and IV in the electron transport chain) [37]. By continuously pumping hydrogen ions into the intermembrane space, an electrochemical proton gradient accumulates between the outer and inner sides of the IMM, which is the driving force for ATP synthesis [38]. Unfortunately, the electrochemical gradient collapses quickly once the mitochondrial permeability transition pore is opened, which is termed a loss of MMP [39]. In our study, the levels of MMP and ATP were decreased after irradiation, which is consistent with our previous study [12], whereas both were well maintained by salidroside in irradiated SMG cells, suggesting a protective role of salidroside in preventing radiation-induced mitochondrial dysfunction.

**Figure 7:**
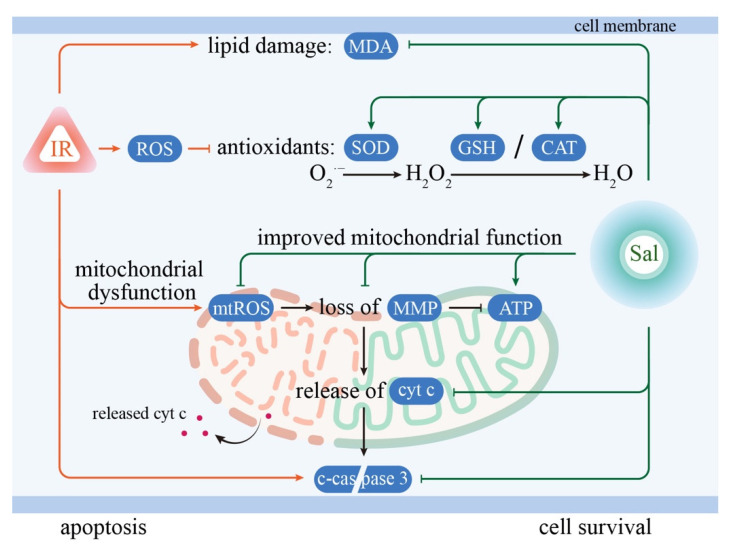
Schematic diagram illustrating the underlying mechanisms by which salidroside improves the survival of irradiated SMG cells against radiation damage by inhibiting oxidative injury at the mitochondrial level. Radiation induced oxidative stress damages in SMG cells, including by increasing MDA, decreasing SOD, CAT, and GSH, enhancing mitochondrial ROS, and inducing cell apoptosis. In contrast, salidroside improved the recovery of the mitochondrial ultrastructure and energy metabolism by scavenging mitochondrial ROS and improving antioxidant defense ability, which eventually led to the inhibition of cellular apoptosis in irradiated SMG cells. Abbreviations: IR, ionizing radiation; Sal, salidroside; ROS, reactive oxygen species; MDA, malondialdehyde; SOD, superoxide dismutase; CAT, catalase; GSH, glutathione; mtROS, mitochondrial ROS; MMP, mitochondrial membrane potential; ATP, adenosine triphosphate; cyt c, cytochrome c; c-caspase 3, cleaved-caspase 3.

Mitochondrial-related cell apoptosis may represent the key mechanism underlying radiation-induced hyposalivation in the early phase [40]. Mitochondrial disruption causes the collapse of MMP, which in turn initiates the release of cytochrome c and downstream apoptotic events [41]. In the present study, our data showed that radiation induced a sharp increase in cytosolic cytochrome c and cleaved-caspase 3 in irradiated SMG cells; however, both were significantly decreased by the salidroside pretreatment. Concomitant with the above alterations in mitochondrial-related apoptosis proteins, the number of apoptotic cells was significantly increased in irradiated SMG cells and significantly reduced by salidroside pretreatment. These findings demonstrate that salidroside protects SMGs against mitochondrial-related apoptosis after irradiation. The mechanisms underlying the ability of salidroside to protect SMGs against radiation are depicted in Figure 7.

Mitochondria are believed to be early responders and vital players in radiation-induced dysfunction of salivary glands [33]. Generally, the greatest morphological changes are observed from Days 2 to 4 after irradiation, with some quality restoration emerging from Day 10 after irradiation [42]. Moreover, the secretion function of SMGs is decreased by approximately 50% at Day 3 after irradiation with 15 Gy [42,43,44]. A novel study showed that MitoTEMPO induced a significant recovery of secretion function in mice irradiated salivary glands, suggesting that inhibiting the initial increase in mitochondrial ROS by MitoTEMPO induces obvious protection of salivary gland secretion function after irradiation [33]. Interestingly, our data demonstrated that salidroside not only protected the histostructure of SMGs from radiation damage but also significantly promoted the secretion function of rat SMGs after irradiation, and the underlying mechanisms may be associated with scavenging mitochondrial ROS by salidroside. Moreover, the sterilization of salivary gland stem/progenitor cells induced by radiation contributes to the later phase (120–240 days post irradiation) of hyposalivation [45]. Future studies should investigate the roles and underlying mechanisms of salidroside in improving the regeneration of stem/progenitor cells in the irradiated salivary gland at the late phase.

Based on literature reports, salidroside has contrasting effects on normal and cancer cells. In normal cells, salidroside plays important roles in anti-inflammation, anti-stress, antioxidant, anti-aging, and anti-viral activities [46], while in cancer cells, salidroside inhibits cell proliferation and induces cell apoptosis, including urinary bladder cancer, breast cancer, gastric and colorectal cancer, lung cancer, nasopharyngeal carcinoma (NPC), and sarcoma [46,47]. Meanwhile, salidroside had no cytotoxic effect on the normal cells, such as thyroid follicular epithelial cells and human mammary epithelial cells, when it inhibited the cellular migration and invasion in poorly differentiated thyroid cancer cells or breast cancer cells with the drug concentrations of 5~40 µM [48,49]. Furthermore, salidroside could inhibit the growth of NPC xenografts, transplanted solid Ehrlich adenocarcinoma, and Pliss lymphosarcoma [47,50]. Taken together, salidroside exhibits significant anti-cancer efficacies both in in vivo and in vitro experimental studies. Therefore, salidroside may not reduce radiation’s efficacy in killing cancer cells. The exact pharmacological characteristics of salidroside in head and neck cancers need further investigation in an animal tumor model.

## 5. Conclusions

In conclusion, excessive ROS in salivary glands induced by radiation commonly leads to damage to the mitochondrial structure and functions, consequently resulting in cell apoptosis and dysfunction of salivary glands. Our findings demonstrate that salidroside protects SMG cells and tissues from radiation. Importantly, salidroside inhibits mitochondrial-related apoptosis by scavenging mitochondrial ROS, enhancing antioxidant defense, and preserving mitochondrial structure and functions from radiation-induced damage in SMGs, thereby promoting the secretion function. Overall, these findings identify salidroside as a mitochondrial-targeted antioxidant for preventing radiation-induced damage to salivary glands, and they suggest that the application of salidroside can be a safe and effective strategy for patients suffering from radiation sialadenitis or other oxidative stress-related salivary gland dysfunction.

## Data Availability

The data presented in the study are all contained within this manuscript.
